# Continuous gas-phase synthesis of core–shell nanoparticles *via* surface segregation†

**DOI:** 10.1039/d0na01061h

**Published:** 2021-04-14

**Authors:** Markus Snellman, Namsoon Eom, Martin Ek, Maria E. Messing, Knut Deppert

**Affiliations:** Lund University, Department of Physics and NanoLund Box 118 22100 Lund Sweden knut.deppert@ftf.lth.se; Lund University, Department of Chemistry and NanoLund, Centre for Analysis and Synthesis Box 124 22100 Lund Sweden

## Abstract

Synthesis methods of highly functional core@shell nanoparticles with high throughput and high purity are in great demand for applications, including catalysis and optoelectronics. Traditionally chemical synthesis has been widely explored, but recently, gas-phase methods have attracted attention since such methods can provide a more flexible choice of materials and altogether avoid solvents. Here, we demonstrate that Cu@Ag core–shell nanoparticles with well-controlled size and compositional variance can be generated *via* surface segregation using spark ablation with an additional heating step, which is a continuous gas-phase process. The characterization of the nanoparticles reveals that the Cu–Ag agglomerates generated by spark ablation adopt core–shell or quasi-Janus structures depending on the compaction temperature used to transform the agglomerates into spherical particles. Molecular dynamics (MD) simulations verify that the structural evolution is caused by heat-induced surface segregation. With the incorporated heat treatment that acts as an annealing and equilibrium cooling step after the initial nucleation and growth processes in the spark ablation, the presented method is suitable for creating nanoparticles with both uniform size and composition and uniform bimetallic configuration. We confirm the compositional uniformity between particles by analyzing compositional variance of individual particles rather than presenting an ensemble-average of many particles. This gas-phase synthesis method can be employed for generating other bi- or multi-metallic nanoparticles with the predicted configuration of the structure from the surface energy and atomic size of the elements.

## Abstract 2

A continuous gas-phase synthesis method is employed to generate Cu@Ag core–shell nanoparticles with uniform size, composition, and bimetallic configuration. We discuss how the bimetallic morphology can also be tuned into a quasi-Janus structure.

## Graphical abstract

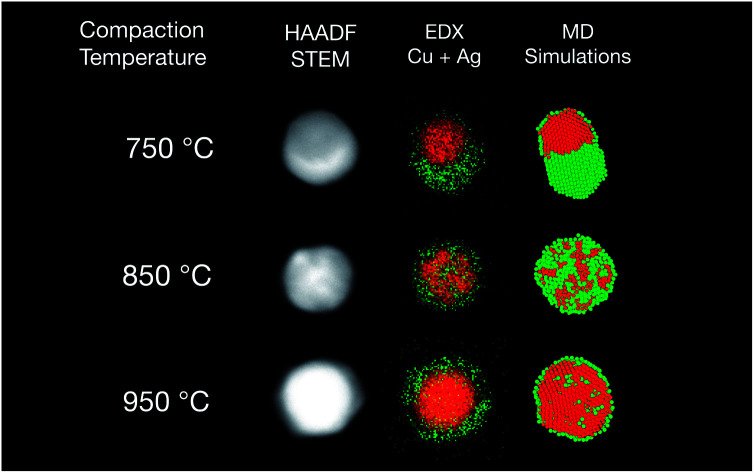

## 1. Introduction

Recently, a significant amount of research effort has been devoted to the production of core–shell nanoparticles, which are composed of an inner core material coated by a shell of a different material. Such attention to core–shell nanoparticles arises from the fact that they can exhibit enhanced physical and/or chemical properties.^1–3^ Furthermore, core–shell particles with distinctly new properties compared to those of the constituent materials can be designed by tuning, for example, their size, shell thickness, and structures.^4–7^ A large number of research projects are underway to fabricate highly functional core–shell materials for applications in various fields, including optoelectronic devices,^8,9^ biomedical imaging,^10,11^ catalysis,^12,13^ and plasmonics.^14,15^

Currently, chemical synthesis techniques such as sol–gel,^16,17^ solvothermal,^18^ seed-mediated growth,^19^ and cation exchange^20^ are the most popular methods for fabricating core–shell nanoparticles. However, interface and surface contaminations are often an unavoidable issue in the multiple-step, solution-based approaches. These impurities inevitably make solution-based processes time consuming as many steps are required to remove contaminants. The process of removing ligands also introduces uncertainty regarding the final size and structure of the nanoparticles.^21,22^ Contrary to the widely popular chemical synthesis, significantly less attention has been paid to solvent-free gas-phase synthesis methods, which offer high purity and high throughput in nanoparticle production. Recently, gas-phase synthesis techniques based on low-pressure multi-magnetron gas aggregation sources – where one target acts as the source of the core material and the others as one or more sources coating materials – have enabled the fabrication of core–shell particles with tunable sizes and shapes.^23,24^ Apart from the demanding high vacuum requirements,^25^ these methods often suffer from nucleation of pure-element byproducts,^24–26^ and achieving uniformity in bimetallic morphology is challenging as the nanoparticles generated by non-equilibrium, fast kinetics processes that do not include an additional annealing process often include random and unpredictable metastable phases.^27,28^ Having control over size, composition, and morphology is desirable, as it enables investigations of the nanoparticle properties' effects on various applications. We note that in this article we use the term ‘metastable’ for any nanoparticle configurations that are not in the thermodynamically stable global energy minimum.

Here, we present a continuous gas-phase process based on spark ablation^29^ with the capability of creating uniformly structured core–shell bimetallic nanoparticles with precisely controlled size and composition not containing other random metastable configurations. Spark ablation is a gas-phase synthesis technique with an appealingly simple design that utilizes a high voltage spark discharge between two electrodes acting as the material source for the synthesized nanoparticles. It has been used to create various types of materials such as semiconducting nanoparticles^30^ and composite metal nanoparticles.^31^ Similar to a related technique known as arc discharge,^32–35^ it is an environmentally-friendly, inexpensive alternative to chemical synthesis techniques and offers a continuous production route at atmospheric pressure. The technique can readily be upscaled to mass-production by placing several electrode pairs in parallel. Recently, spark ablation has been used to produce Ag@Au and Au@Ag core–shell nanoparticles *via* a condensation mechanism which requires modification of the conventional spark ablation for a separate coating step.^36^

In this study, we exploit the surface segregation phenomenon in generating core–shell bimetallic nanoparticles using spark ablation in a continuous process without an additional coating step. The surface segregation phenomenon refers to the enrichment of one component of a mixture in the surface region. It is generally agreed that surface segregation depends on an interplay between the atomic radii, cohesive energy, surface energy, and electronegativity of the core and shell materials.^37^ As a rule of thumb, one expects that the metals with smaller atomic radii and larger surface energies would tend to occupy the core region. Utilizing the surface segregation mechanism, one can produce core–shell nanostructures by simply evaporating both core and shell materials simultaneously in the gas phase, rendering the process ‘continuous’ without the need of a separate coating process.^38–42^ Note that *in situ* heat treatment in gas phase synthesis methods has been reported to be efficient for phase transformation.^43^ In our setup, heat-induced surface segregation occurs when the agglomerates of bimetallic nanoparticles, synthesized by spark ablation, pass through a tube furnace during which the agglomerates become spherical core–shell structures.

In generating bimetallic core–shell nanoparticles using spark ablation *via* surface segregation, we have chosen Cu–Ag as our model system as the atomic radius mismatch is relatively high, and the surface energy difference is sufficient (1210 mJ m^−2^ for Ag and 2130 mJ m^−2^ for Cu).^44^ Additionally, Cu–Ag is a well-studied immiscible material system. We have investigated the morphology, composition, and inter-particle heterogeneity of the generated Cu@Ag core–shell nanoparticles by scanning transmission electron microscopy (STEM), and energy-dispersive X-ray spectroscopy (EDX). To provide more in-depth insight into the structural evolution of Cu–Ag nanoparticles during the heating and cooling processes, we also conducted molecular dynamics (MD) simulations. The numerical modeling corroborates our experimental results that the compaction temperature influences the nanoparticle's final structure and that the core–shell formation is attributed to the heat-induced surface segregation.

In addition to the capability of generating uniform core–shell nanoparticles with well-controlled size and composition, exploiting the surface segregation together with the spark ablation method is further beneficial. As the core@shell nanoparticles generated *via* the presented synthesis method have already undergone a heating cycle, they are expected to exhibit high structural stabilities at elevated temperatures, which is supported by the MD simulations. It is well-known that nanoparticles with equilibrium shape and narrow-sized distributions are favorable for suppressing sintering.^45^ The suppression of sintering makes the method appealing for catalysis applications, where the structural stability of bimetallic nanoparticles in elevated temperatures is essential.

The gas-phase synthesis method presented here can be employed for other bi- or multi-metallic systems with sufficient differences in surface energy and atomic radius of the elements for generating core–shell nanoparticles. However, this method is not just limited to the production of core–shell nanoparticles. The same method can also be used to create other structures (*e.g.*, quasi-Janus or alloy) with the only requirement to design the desired structures being the knowledge of the surface energy and atomic size of the constituent elements.

## 2. Experimental

### 2.1. Nanoparticle generation

First, the spark ablation system (Fig. S1†), where spark ablation takes place, was evacuated with a rotary pump. After reaching a pressure of lower than 1 mBar, N_2_ : H_2_ carrier gas (95% : 5%; purity 99.9999%; Linde) was let in at a flow rate of 1.68 L min^−1^, regulated with mass flow controllers (Bronkhorst, El-Flow-Select) and the pressure was kept at 1015 mBar with a pressure controller (Bronkhorst, El-Press-Select). A high-voltage, high power supply (Technix, Model CCR15-P-750) was used to charge a 20 nF capacitor bank shunted to the metallic electrodes enclosed in a chamber flushed with the carrier gas. The electrodes are separated by an air gap of about 2 mm. A grounded pure Cu electrode (GoodFellow, >99.99%) and a biased pure Ag electrode (GoodFellow, >99.95%) were used in this work. At a specific voltage over the capacitors and electrode gap, the carrier gas in the electrode gap breaks down into a conducting plasma, carrying an oscillating current from the discharging capacitor bank that ablates material from the electrodes' surfaces. The ablated material vapors nucleate into small (<10 nm) singlet nanoparticles that grow by full coalescence from collisions until they reach a diameter where further collisions between the primary particles lead to the formation of fractal-like agglomerates by coagulation and partial sintering.^46^ After a few ms, the electrode gap regains its resistive properties, and the charge cycle is repeated. The breakdown voltage was monitored and set to 3.0–4.0 kV implicitly by the electrode gap.

### 2.2. Nanoparticle compaction and size selection

After generation, the Cu–Ag agglomerates were carried by the carrier gas downstream in the setup for subsequent thermal treatment (compaction) and size selection. First, the particles were assigned a known charge distribution in a β emitting ^63^Ni neutralizer. This enables subsequent size selection *via* electrical mobility using a tandem differential mobility analyzer (DMA) setup,^47^ consisting of two DMAs (DMA1: TSI 3081 Long; DMA2: custom Vienna type^48^) with 10 L min^−1^ sheath flows. Inside the DMAs, an electric field classifies particles of particular electrical mobility (mobility in an electric field), a function of diameter and charge. The agglomerates were compacted to spherical particles in a tube furnace (Lenton LTF) positioned between DMA1 and DMA2. After size selection in DMA2, the particles were either counted with an electrometer (TSI 3086B) or deposited with an electric field in a custom electrostatic precipitator (ESP).^49–51^ The electrical mobility range of selected particles is proportional to *Q*_a_/*Q*_sh_ where is *Q*_a_ is the carrier gas flow rate (1.68 L min^−1^) and *Q*_sh_ is the DMA sheath flow rate (10 L min^−1^), equivalent to a flow ratio of *ca.* 1/6 and a size distribution width of the size selected aerosol nanoparticles with a diameter *D*_p_ of ±1/6 *D*_p_.^52^ Using the tandem DMA setup, even narrower size distributions can be obtained, as shown in Fig. S2† where compacted CuAg aerosol nanoparticles were deposited with an electrical mobility diameter of 30 nm and measured from SEM micrographs.

### 2.3. Particle characterization

Electron microscopy and elemental analysis were used to characterize Cu@Ag nanoparticles offline. Only DMA2 was used (DMA1 was bypassed) to ensure a shorter deposition time at the cost of a broader particle size distribution. Particles size selected with DMA2 at electrical mobility diameter of 30 nm were deposited in the ESP set to 6–10 kV on holey carbon film coated Au TEM grids (Agar Scientific). The ESP chamber was purged in N_2_ (purity 99.999%) before and after deposition. Depositing 30 nm particles ensured a sufficient stability necessary for acquiring STEM-EDX maps with enough counts for detailed analysis. The samples were handled in air before STEM imaging (JEOL 3000F operated at 300 kV). In STEM mode, the particles were imaged with a high-angle annular dark-field (HAADF) detector. Elemental distribution maps (EDX maps) were also obtained in STEM mode with the coupled EDX spectrometer (Oxford Instruments), and the data was analyzed and processed in the INCA software (Oxford Instruments) and with the Hyperspy package^53^ in Python to extract the composition of Ag-rich and Cu-rich phases. Additional TEM-EDX statistics on 30 single Cu–Ag nanoparticles per furnace temperature at temperatures of 750 °C, 850 °C and 950 °C were acquired in a 300 kV Hitachi TEM, with similar EDX spectrometer, and the data was analyzed with the associated Aztec software (Oxford Instruments).

### 2.4. Molecular dynamics simulations

Embedded-atom method (EAM)^54^ potentials for the Cu–Ag system developed by Williams *et al.*^55^ were employed in the molecular dynamics simulations. Spherical Cu and Ag nanoparticles were constructed from their perfect face-centered cubic (FCC) crystals with particle diameters ranging from 2.5 to 4.2 nm. The unsupported Cu and Ag nanoparticles were equilibrated separately at 27 °C for 100 ps and were subsequently placed next to each other in a nonperiodic vacuum cell. This initial relaxation process leads to a Cu–Ag aggregate that is a small representation of metastable aggregate created from the fast quenching process in spark ablation. The equations of motion were integrated by the velocity-Verlet algorithm^56^ with a time step of 1 fs. To simulate the compaction process in the furnace, the aggregate of Cu and Ag were continuously heated up to 750 °C, 850 °C, and 950 °C at a heating rate of 0.13 °C ps^−1^. The system was then cooled at a cooling rate of 0.13 °C ps^−1^ and equilibrated for 100 ps once the temperature reached 27 °C. Note that a combination of MD and Monte Carlo (force-bias method) simulations was employed for the nanoparticle compacted at 850 °C as its structure did not reach a crystalline state in the MD simulation. The canonical ensemble (*i.e.*, NVT) was employed with Nosé–Hoover thermostat for temperature control. Additional simulations for larger particles (6 nm and 10 nm in diameter) were carried out under the same simulation setup. All the simulations were performed using the LAMMPS^57^ code, and PyMOL^58^ and OVITO^59^ were used for visualizations. Crystallinity of the simulated nanoparticles were analyzed using polyhedral template matching.^59,60^

## 3. Results and discussion

### 3.1 Compaction behavior of the Cu–Ag nanoparticles

The particles generated in the spark-discharge chamber are fractal-like agglomerates consisting of primary particles in the size of 2–10 nm. It has been reported that Cu–Ag nanoparticles generated from spark ablation of sintered Cu–Ag electrodes show an increase in Cu–Ag solubility on the nanoscale despite their intrinsic immiscibility.^61^ Similarly, it has been shown that primary particles generated from two different immiscible metal electrodes can form mixed crystalline phases given a sufficiently fast quenching.^62^ Thus, although it is challenging to correctly characterize the composition and morphology of primary particles due to their small size, we believe that there is a high likelihood that the primary particles in the Cu–Ag nanoparticle agglomerates created in the spark ablation are binary mixtures of Cu and Ag. And, as the agglomerates approach the tube furnace, segregation is expected to take place due to increased atomic diffusion in the heating process. We discuss this further in the later section.

**Fig. 1:**
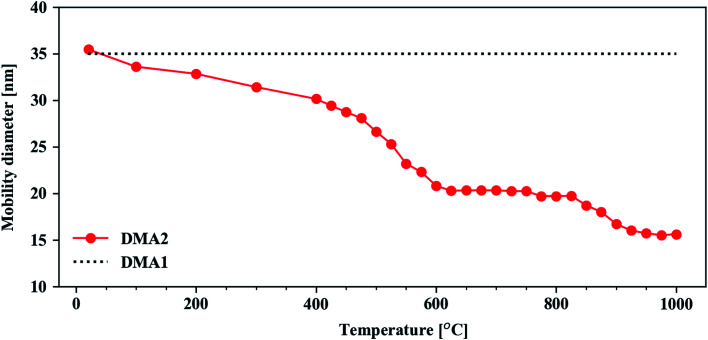
Particle characterization in the aerosol phase. The Cu@Ag compaction behavior was obtained using the tandem DMA setup. The line connecting the dots is a guide for the eye.

The Cu–Ag particle agglomerates become compacted to spherical particles as they pass through the tube furnace. By keeping DMA1 at a fixed electric mobility diameter of 35 nm and scanning the electric mobility diameter of DMA2, the compaction behavior of the Cu@Ag nanoparticles was obtained in a tube furnace temperature range from room temperature to 1000 °C (Fig. 1). Each data point in Fig. 1 corresponds to the electric mobility diameter associated with the maximum particle concentration for that temperature.

**Fig. 2:**
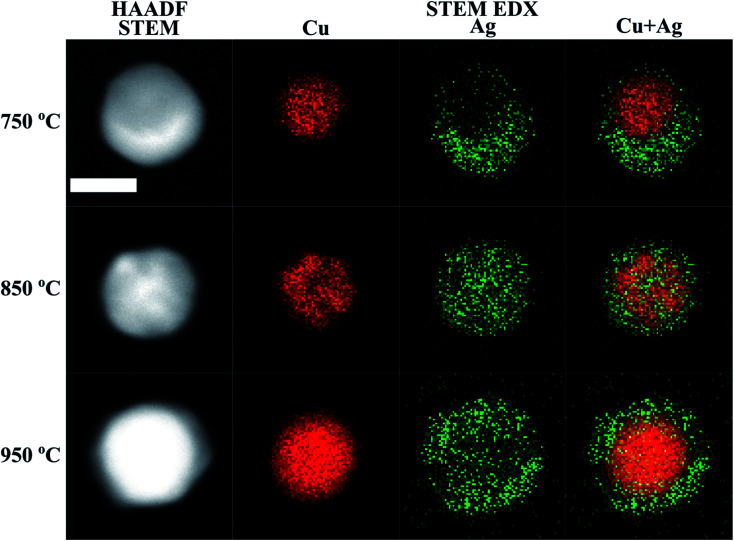
HAADF STEM micrographs, and Cu, Ag and Cu + Ag overlaid STEM-EDX maps of Cu@Ag nanoparticles compacted at different temperatures. The scale bar is 20 nm for all micrographs and maps.

When the furnace was set to room temperature, the mobility diameter of the nanoparticles scanned by DMA2 coincides with that of DMA1. This implies that there is no morphological change in the nanoparticles at that temperature. As the furnace temperature increases, however, the mobility diameter decreases with more noticeable changes at 400–600 °C. At this temperature range, the structural evolution from a clustered, fractal particle morphology to a fully compacted, spherical particle takes place.^63–65^ Spherical particles are observed at *ca.* 600 °C, which is congruent with previously observed compaction temperatures of metallic aerosol nanoparticles at 1/3–2/3 of the bulk melting temperature in kelvin,^66^ which for Cu and Ag are 1357.75 K (1084.6 °C) and 1234.95 K (961.8 °C), respectively.^67^ At higher temperatures (>600 °C), little to no further compaction occurs as indicated by the first plateau at 600–800 °C. This is supported by the spherical morphology of the Cu@Ag particles in the STEM-EDX maps at different temperatures shown in Fig. 2. Although the Cu@Ag particles become more or less spherical at 600 °C, internal restructuring processes are expected to continue in the particles at higher temperatures.^66,68^ Over the range of about 900–950 °C, a transition to a second mobility diameter plateau occurs. We attribute this transition to increased Ag evaporation and depletion from the surface of the particles. A high volatility of Ag in nanostructure has previously been reported from *in situ* STEM studies by Lu *et al.*^69^ Additionally, the Ag depletion is corroborated qualitatively by evaporation rates predicted by the Knudsen equation (SE1), as plotted in Fig. S3 in ESI.† Above 1000 °C, we expect the material evaporation rate of both Ag and Cu to increase further and hence a continued reduction in the electric mobility diameter in Fig. 1. The consequent Cu enrichment will be discussed further in connection to the compositional analysis in the following section.

### 3.2 Morphology and composition of the Cu–Ag nanoparticles

STEM-EDX maps of single nanoparticles compacted at three different temperatures (750 °C, 850 °C and 950 °C) were obtained to determine the Cu and Ag distribution and are shown in Fig. 2. We observe two distinct morphological phases of Cu@Ag nanoparticles in the STEM-EDX maps. When compacted at 750 °C, the particles adopt a quasi-Janus structure. However, at 850 °C, the EDX maps clearly indicate a core@shell morphology. In all the STEM-EDX maps in Fig. 2, it appears that Ag is present in Cu rich parts and *vice versa*. Using non-negative matrix factorization (NMF) with the Python library Hyperspy,^53^ we were able to separate the EDX maps into Cu rich and Ag rich components for particles synthesized at 750 °C, 850 °C and 950 °C for detailed quantification of the Cu rich and Ag rich segments (Fig. S4–S6†). The atomic composition in the Cu rich and Ag rich parts of particles synthesized at 750 °C (quasi-Janus particles), 850 °C and 950 °C (core@shell particles) are shown in Table 1. Moreover, the NMF spectral components in Fig. S4–S6† reveal little to no oxygen in the Cu rich cores, while a small oxygen signal was detected in the Ag rich shell for the particles compacted at 850 °C and 950 °C (Fig. S5 and S6†). This signifies a resistance to oxidation, possibly due to high surface content of Ag. Although the samples were handled in air prior to STEM-EDX, the addition of 5% H_2_ to the carrier gas has been shown to be beneficial for reducing oxidation of particles synthesized by SDG.^70^ We detected clear signs of oxidation only after several weeks of ambient storage.

**Table 1:** STEM-EDX composition of Cu rich and Ag rich parts of Cu@Ag nanoparticles determined from four particles (750 °C, 850 °C) and three particles (950 °C)

	750 °C		850 °C		950 °C	
	Cu-rich phase	Ag-rich phase	Cu core	Ag shell	Cu core	Ag shell
Cu (at%)	97.9 ± 3.0	11.7 ± 2.2	89.4 ± 3.5	11.8 ± 1.5	99.9 ± 0.1	28.0 ± 8.1
Ag (at%)	2.1 ± 3.0	88.3 ± 2.2	10.6 ± 3.5	88.2 ± 1.5	0.1 ± 0.1	72.0 ± 8.1

**Fig. 3:**
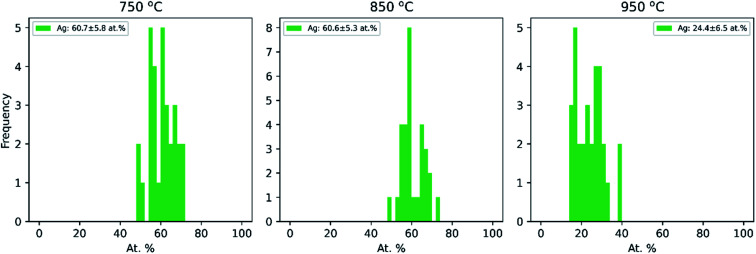
Ag composition distribution for Cu–Ag particles as determined by TEM-EDX for 30 individual particles at each temperature.

Additionally, the average compositions, as determined by TEM-EDX of 30 particles per temperature at the same three temperatures (750 °C, 850 °C and 950 °C), are given in Fig. 3. It shows that the Cu–Ag particle composition range is narrow at all three temperatures, with a standard deviation of 5–7 at%. Clearly, the particles become enriched with Cu at 950 °C, which correlates well with the decrease in mobility diameter observed in the compaction study above 900 °C (Fig. 1), that was attributed to Ag evaporation. Typically, the composition of gas phase synthesized nanoparticles is investigated by interrogating a large number of particles simultaneously.^25,26,71,72^ This approach provides a good sample of ensemble-averaged properties of many particles, but cannot provide information on compositional variance between particles. Indeed, to the best of our knowledge, the compositional uniformity between individual bimetallic nanoparticles synthesized from coagulating and/or coalescing monometallic particles in the gas-phase is not well-documented. Krishnan *et al.*^73^ reported a very low inter-particle compositional variance on single nanoparticles synthesized by a sectional Mo–Cu sputtering target, but did not account for the number of particles interrogated by EDX. This is to the best of our knowledge the first time that compositional variance of individual particles synthesized by sintering of agglomerates formed by coagulation of bimetallic species has been studied, and is of relevance for multiple gas phase based techniques for synthesis of bimetallic nanoparticles from coagulating and coalescing particles. A quasi-Janus or crescent morphology observed at a compaction temperature of 750 °C has been previously reported for this material system^74–76^ although not for Cu@Ag particles synthesized in the gas phase to the best of our knowledge. Langlois *et al.*^75^ studied the annealing of Cu@Ag core–shell nanoparticles on a substrate and observed that the structure transformed to Janus-like quasi-Janus when the amount of Ag in a particle is large. They reported a quasi-Janus configuration adopted beyond a critical Ag shell thickness of 3–4 nm. A global optimization study on small (100 to 300 atoms) Cu–Ag particles of varying composition supported on MgO(001) also showed the preference of Ag to migrate to the surface, with quasi-Janus morphologies appearing at higher Ag concentrations.^77^ Comparing with our results in Fig. 2 and 3, a simple geometrical derivation for a spherical core–shell particle of uniform core and shell compositions (eqn (E6) in ESI†) suggests an Ag shell thickness of *ca.* 4.9 nm, 4.8 nm and 1.8 nm for the particles compacted at 750 °C, 850 °C and 950 °C, respectively. In our study, we do not find a clear relation between the element quantity and particle morphology, and hence the observation made by Langlois *et al.* is not supported by our experiments. The particles studied by that group^75^ were, however, synthesized in a fundamentally different way *via* evaporation and thermal dewetting, where the substrate may play a significant role in the formation and the thermodynamical stability of the particles. In our study, where Cu–Ag agglomerates compact directly in the gas phase, we determine the compaction temperature to be the most crucial variable in deciding the morphology of Cu@Ag core–shell nanoparticles.

Numerous theoretical works have been reported on the phase stability of Janus, core–shell, and alloyed Cu–Ag nanoparticles with differing conclusions. A model based on classical thermodynamics predicts the Janus morphology for nanoparticles with small Cu core size and large Ag quantity.^78^ The same model, however, also predicts the preference of an alloyed composition over the core–shell morphology for particles with size and composition similar to those synthesized here, which we do not observe. Another thermodynamical model based on surface energy differences of Ag and Cu,^76^ where the authors synthesized crescent (quasi-Janus) and Cu@Ag core–shell nanoparticles by a solution process, suggests that a quasi-Janus morphology is always preferred but that the energetic difference between the two morphologies decreases with increasing Ag content, making the core@shell morphology more likely for particles with a high Ag fraction. This proposed trend is not reflected in our results as particles synthesized at 750 °C and 850 °C have a similar composition yet a different morphology, *i.e.*, the particles compacted at 750 °C adopt a quasi-Janus structure, while the particles compacted at 850 °C adopt a core@shell morphology. Additionally, the particles compacted 950 °C also adopt a core@shell morphology with significantly less Ag content (<25 at%). We note that the model proposed by Osowiecki *et al.*^76^ should be accompanied by modeling of the significant strain energy present in the Cu–Ag interface, due to the lattice mismatch of 13%,^78,79^ such as the continuum-mechanics model implemented for CdTe@CdSe^80^ and CdSe@CdS^81^ colloidal core@shell nanoparticles. The model in ref. 76 is further complicated by the surface energy dependence on particle diameter. While several models predict a decrease of surface energy with decreasing nanoparticle radius, as compared to bulk values,^82,83^ experimental data on nanoparticles suspended in the gas-phase show opposite tendency.^84,85^ Hence, it is clear that a suitable model for synthesis of core–shell structures *via* equilibrium gas-phase processes is lacking, which we address in the next section.

### 3.3 Molecular dynamics simulations

We performed molecular dynamics simulations to obtain further insight into the structural evolution of Cu–Ag nanoparticles during the heating and cooling processes. We carried out simulations based on well-established MD-routines for the Cu–Ag nanoparticle system,^27,86–88^ to demonstrate how quasi-Janus and core–shell structures can form from an aggregate by adjusting only compaction temperature. We mimic the experimental conditions by including both heating and cooling process that corresponds to entering and exiting the tube furnace.

First, we present the simulation results and analysis of small particles (∼4 nm in diameter). In order to employ the MD results in explaining the experimental results on significantly larger particles, later in the section, we discuss the simulation results on larger particles (6 nm and 10 nm in diameter).

The TEM-EDX analysis shows that the atomic percentage of the Cu–Ag nanoparticles compacted at 750 °C was Cu : Ag = 39 : 61 (see Fig. 3). For this atomic ratio, the compaction between a Cu nanoparticle with a diameter of 3.0 nm and an Ag nanoparticle with a diameter of 3.9 nm was simulated.

**Fig. 4:**
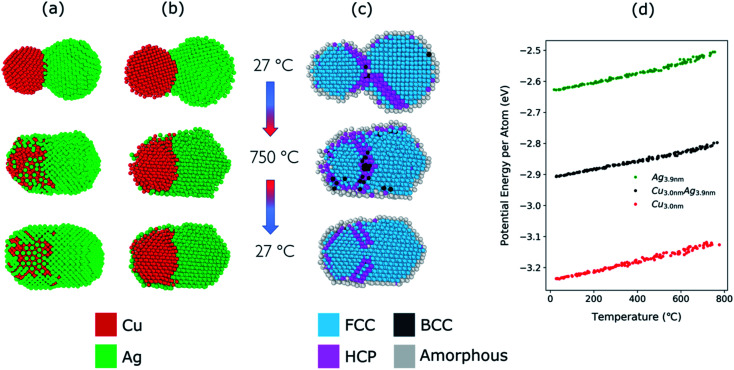
(a) Structural evolution of Cu(red)–Ag(green) nanoparticles when heated from 27 °C to 750 °C and cooled back down to 27 °C. Here, the atomic ratio of the Cu–Ag is Cu : Ag = 39 : 61. (b) Cross-sectional view of the simulated nanoparticles. (c) Cross-sectional view of the crystallinity of the simulated nanoparticles during the heating and cooling. (d) Potential energy per atom of Cu (3.0 nm), Ag (3.9 nm), and Cu–Ag nanoparticles during the heating process.

The structural evolution of a Cu–Ag nanoparticle at this temperature is shown in Fig. 4a and b, along with the evolution of crystallinity (Fig. 4c) and the change in the average potential energy per atom (Fig. 4d). As the particles are heated from 27 °C to 750 °C, surface atoms of Ag start diffusing to the surface of Cu as expected from the lower surface energy and cohesive energy of Ag. It was observed that the Ag atoms do not readily diffuse into the Cu core region. At 750 °C, the system forms a quasi-Janus structure with Ag atoms on the surface of Cu. As the temperature decreases, the Janus structure remains unchanged except for the continued diffusion of Ag atoms on the Cu surface. When the system is cooled back to a temperature of 27 °C, an overall crystalline quasi-Janus Cu–Ag nanoparticle forms. A few Cu atoms diffuse to the Ag side. This overall quasi-Janus morphology in the MD simulation agrees with the STEM EDX maps (Fig. 2) of the Cu–Ag nanoparticles compacted at the same temperature. It is further supported by the quantification of the NMF of the same EDX maps (Table 1), where the Cu-rich phase contains little Ag and some Cu have incorporated in the Ag-rich phase. While an increased solubility of Cu in Ag has been observed previously upon quenching Cu–Ag mixed nanoparticles in inert gas condensation,^89^ this is, to the best of our knowledge, the first time it has been observed in a comparatively slower cooling cycle.

To determine the melting point of the simulated Cu–Ag nanoparticles, the average potential energy per atom as a function of the temperature is plotted (Fig. 4d). Additionally, MD simulations for single-particles are conducted on a single Cu and a single Ag particle to obtain references for the melting behavior of Cu–Ag bimetallic particles (Fig. 4d). The melting point is generally defined to be the temperature at which the potential energy increases abruptly due to the absorption of latent heat of fusion.^90,91^ It is well known that the melting point of nanoparticles is size-dependent, that is, it decreases as the size of nanoparticle decreases.^92^Fig. 4d shows the change in potential energy of a 3.0 nm single Cu, and of a 3.9 nm single Ag nanoparticle. The average potential energy per atom increases linearly with increasing temperature. In Fig. 4d, the potential energy per atom of both Cu and Ag do not show any abrupt jumps. This implies that at 750 °C, neither Cu nor Ag melts. Crystallinity analysis^59,60^ of the MD results also indicates that each nanocluster remains crystalline during the heating and cooling as shown in Fig. 4c. It is clearly seen that at 750 °C, the crystallinity of both Cu and Ag remain FCC during the coalescence. Thus, it supports an assumption that the quasi-Janus structure is created by surface diffusion.

**Fig. 5:**
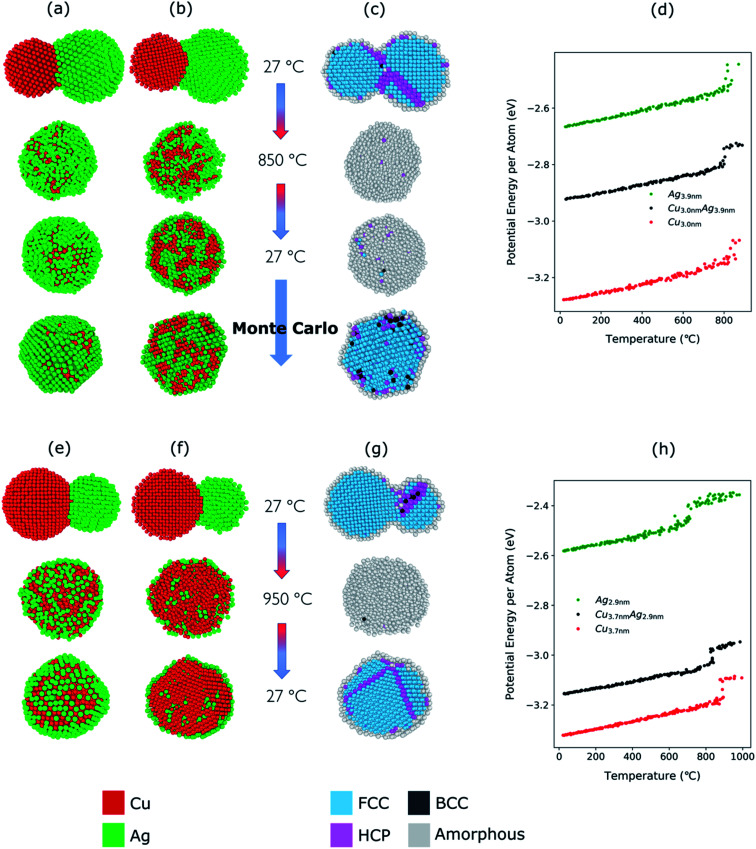
(a) Structural evolution of Cu(red)–Ag(green) nanoparticles when heated from 27 °C to 850 °C and cooled back down to 27 °C. Here, the atomic ratio of the Cu and Ag is Cu : Ag = 39 : 61. (b) Cross-sectional view of the simulated nanoparticles. Note that Monte Carlo was used to obtain the crystalline structure of the nanoparticle at room temperature (c) cross-sectional view of the evolution of crystallinity (d) potential energy per atom of Cu (3.0 nm), Ag (3.9 nm), and Cu–Ag nanoparticles during the heating process. The same analysis is shown in (e–h) as in (a–d) but for a furnace temperature of 950 °C with the atomic ratio of Cu and Ag, Cu : Ag = 76 : 24. (h) Potential energy per atom of Cu (3.7 nm), Ag (2.9 nm), and Cu–Ag nanoparticles during the heating process.

For the Cu–Ag nanoparticles compacted at 850 °C with an atomic ratio of Cu : Ag = 39 : 61 (see Fig. 3), a Cu nanoparticle with a diameter of 3.0 nm and an Ag nanoparticle with a diameter of 3.9 nm were simulated and are shown in Fig. 5a–d. At 850 °C, both the single Ag and the single Cu nanoparticle melt as indicated by the abrupt jump in the average potential energy per atom (Fig. 5d) and a similar jump in the potential energy is also observed for a Cu–Ag nanoparticle. Melting of the nanoparticle at 850 °C was further supported by crystallinity analysis. As shown in Fig. 5c, the initial FCC structures are no longer observed and become amorphous at 850 °C. As the temperature decreases at a cooling rate of 0.13 °C ps^−1^ the Cu–Ag nanoparticle transforms to an internally mixed nanoparticle with an Ag shell. Note that the simulation shows that the mixing in the core is not uniform, *i.e.*, segregation is observed within the core as seen in Fig. 5b, similar to what was observed in another MD study on smaller (2.5 nm) Cu–Ag nanoparticles heated to 327 °C.^93^ This core@shell morphology with a non-uniformly mixed core is consistent with the STEM-EDX observations presented in Fig. 2, and explains the non-spherical and non-homogeneous signal from the Cu core, in contrast to the relatively pure Cu-rich phase and Cu cores observed for nanoparticles compacted at 750 °C and 950 °C, respectively. An additional simulation with a slower cooling rate of 0.0008 °C ps^−1^ (corresponding to 1 μs for cooling) was carried out to investigate the effect of the cooling rate on the mixing state of Cu–Ag system, in other words, to see whether the degree of segregation increases at a slower cooling rate (ESI Fig. S7†). However, even at the slow cooling rate, the nanoparticle contains some Ag atoms in the Cu core, and they are not mixed uniformly with Cu atoms. Regarding the crystallinity, Fig. 5c shows that the final structure of the nanoparticle at room temperature obtained using MD is not crystalline. Thus, we subsequently employed Monte Carlo to determine the crystalline structure of the nanoparticle as presented in Fig. 5c. The Monte Carlo result also agrees with our experimental result that in the bimetallic nanoparticles that are cooled at a much slower cooling rate (in the order of seconds), the Ag content increases in the core compared to the lower compaction temperature. In our results for the core@shell particles synthesized at 850 °C the Cu core contains approximately 10.6% Ag (see Table 1).

The Cu–Ag nanoparticles compacted at 950 °C contain only approximately 24% Ag according to the TEM-EDX analysis (Fig. 3), which we attribute to significant Ag evaporation at that temperature. For the Cu–Ag system with this atomic ratio, compaction between a Cu nanoparticle with a diameter of 3.7 nm and an Ag nanoparticle with a diameter of 2.9 nm was simulated (Fig. 5e–h). Note that the composition of the nanoparticle in the simulation is set to the one we observe in the compacted NP after the presumed Ag evaporation. In other words, the simulation does not include the evaporation process. Fig. 5g and h show that both Cu and Ag melt at this temperature as expected. The 2.9 nm diameter Ag nanoparticle melts at around 750 °C, indicating an apparent melting temperature depression for smaller nanoparticle (Fig. 5h). Patches of monolayer Ag are found on the surface of melted Cu at 950 °C (Fig. 5e). Some Ag atoms diffuse into the core of the Cu. Quantification of the NMF loadings (Table 1) of the corresponding EDX map in Fig. 2 agrees well with the low Ag content in the Cu core observed in the simulation result here. Furthermore, both the simulation and the EDX analysis identify a higher Cu content in the shell compared to the lower compaction temperatures. However, this is likely related to the issue of defining the extent of the very thin shell, leading to the inclusion of some Cu signal from the core. For the EDX maps, the size of the electron probe at the sample is also becoming a limiting factor for singling out the shell for this sample.

We additionally conducted an MD simulation at an intermediate temperature of 790 °C for equally sized Cu and Ag nanoparticles to demonstrate the possibility of optimizing the core–shell morphology and compositions (see ESI Fig. S8†). At this temperature, Ag melts, but Cu does not. Thus, Ag atoms diffuse to the surface of the solid Cu nanoparticle resulting in a core@shell structure without Ag atoms in the core region. This implies that it may be possible to create well-defined Cu@Ag core–shell nanoparticles solely by choosing the right compaction temperature. There is also a possibility that this effect of melting temperature difference between Ag and Cu in nano-regime can lead to quasi-Janus structures. Given the reported high volatility of Ag, one can also assume a significant melting temperature depression of Ag compared to Cu. This would lead to a wide temperature range in which the compacted agglomerate consisted of liquid Ag and solid Cu. In the case of large agglomerates compacted in the experiment, they may form quasi-Janus (or off-center core–shell) structures as the solidification proceeds.

We note that the good agreement observed in the temperatures of the MD simulations and the experimental results in this study is somewhat coincidental. It is well known that the melting point is overestimated in MD simulations due to superheating.^94^ If simulations are performed for larger particles, the melting temperatures will be higher than that of the 4 nm ones, and thus the Janus structures form at higher temperatures (see ESI Fig. S9†). This implies that one needs to be cautious when interpreting MD results for the melting points and the temperatures at which particular morphologies form.

However, the MD results seem to be powerful in eliciting the general trend. The simulation results support that the quasi-Janus and core@shell morphologies observed in the synthesized Cu–Ag nanoparticles at different temperatures are attributed to the immiscibility; combined effect of differences in surface energy, atomic size, and cohesive energies of Cu and Ag nanoparticles.^95^ Even though we discussed the simulation results carried out for small nanoparticles (∼4 nm in diameter), the same trend is observed in simulations performed for larger particles (6 nm and 10 nm in diameter) (ESI Fig. S10†). We observe that regardless of the particle size, quasi-Janus particles are formed at low temperatures, and core@shell particles are formed at high temperatures. Therefore, we are confident that the structural evolution seen in MD simulations can explain the different morphologies observed also for the larger particles in the experiment.

According to Grammatikopoulos *et al.*^27^ who also studied the equilibrium structures of Cu–Ag NPs using combined simulation of MD and Monte Carlo, the quasi-Janus Cu–Ag structure is a metastable state and core–shell-like is an equilibrium state. We have also shown that the equilibrium structure found for the composition investigated in this study (Cu : Ag = 39 : 61) exhibits core–shell-like configurations, *i.e.*, Ag shell with a non-uniformly mixed core. The fact that no quasi-Janus structures were observed at high temperatures in our experiments indicates that quasi-Janus structures are formed mainly by coalescence and surface diffusion of the aggregates at sub-melting temperature. Thus, we conclude that the transition from quasi-Janus to core–shell occurs when the compaction (heat treatment) of the Cu–Ag agglomerates is carried out at higher temperatures. Both the experimental observations and the simulation results point to a likelihood of presence of segregated domains in the nanoparticle aggregates in the tube furnace. Previous research in mixing of primary particles in spark discharge generated agglomerates showed clear alloying in the case of AuPd.^96^ While increased mixing of Cu–Ag primary particles is possible due to the rapid quenching process, segregation likely occurs within individual primary particles as the agglomerates enter the tube furnace. It is noteworthy that this synthesis method can produce bimetallic nanoparticles with different morphology (either quasi-Janus or core–shell) by merely tuning the compaction temperature. The more significant observation is that “uniform” bimetallic nanoparticles with chosen morphology can be readily produced by the presented method. Without a heating step, uniformity is often challenging to achieve with gas-phase synthesis methods that are good for producing various random metastable structures through fast kinetics and non-equilibrium processes.^27,28^ With our synthesis method, we avoid the randomness in the generated nanoparticle morphology by adding the heat treatment process for the Cu–Ag agglomerates.

Another important observation from the MD simulations is that the overall structures of the Cu–Ag nanoparticles remain consistent as they are cooled from high temperatures. This implies that the core–shell bimetallic nanoparticles generated *via* heat-induced surface segregation do not change their overall morphology when treated at high-temperature conditions. This parallels the synthesis method employed in this study where the core–shell nanoparticles generated have already undergone a heating and cooling process, *i.e.*, the heat-induced surface segregation. No reconfiguration of the structure upon heating indicates that the core–shell particles generated *via* the presented method are likely to show high structural stability at elevated temperatures. Structural stability is a critical issue in various applications, especially in catalysis, in which the processes often occur in high-temperature environments. Bimetallic nanoparticles generated by our method are likely to be resistant to a structural transformation upon heating.

## 4. Conclusion

Cu@Ag core–shell nanoparticles have been generated using spark ablation, simply utilizing the surface segregation phenomenon in a continuous gas-phase process. We have demonstrated that the compaction temperature plays a vital role in the creation of the particles. STEM-EDX analysis revealed that the as-generated Cu–Ag agglomerates can be made to adopt quasi-Janus or core–shell structures depending on the compaction temperature. Molecular dynamics simulations support the importance of compaction temperature in deciding the final morphology of the Cu–Ag nanoparticles found in experimental results.

The presented method provides a route of achieving uniformity in core–shell bimetallic nanoparticles in terms of all three aspects; size, composition, and morphology. This is still extremely challenging with other gas-phase synthesis methods involving only non-equilibrium processes without additional annealing and equilibrium cooling processes. The bimetallic nanoparticles produced using our method are expected to exhibit high structural stability when subjected to high-temperature conditions, owing to the compaction process of heating and cooling during the synthesis. We expect that this method is ideal for producing bimetallic nanoparticles for catalysis applications, where the structural stability of nanoparticles in elevated temperatures is of great importance. This simple gas-phase synthesis method is not limited to the production of core–shell nanoparticles but can also be used to create other structures (quasi-Janus, alloy) with high stability. In designing the desired structures, the main properties to consider are the surface energies, atomic radii of the constituent elements, and compaction temperature.

## Conflicts of interest

There are no conflicts to declare.

## Supplementary Material

NA-003-D0NA01061H-s001
