# Supplementary material for: Continuous gas-phase synthesis of core–shell nanoparticles via surface segregation
Source: Nanoscale Adv. 2021 Apr 14;3(11):3041–52. doi: 10.1039/d0na01061h (PMC9419486; doi:10.1039/d0na01061h)
Supplement: NA-003-D0NA01061H-s001 [file NA-003-D0NA01061H-s001.pdf]

Electronic Supplementary Material (ESI) for Nanoscale Advances.
This journal is © The Royal Society of Chemistry 2021

Supporting Information

One-step Gas-phase Synthesis of Core-shell Nanoparticles via Surface Segregation

Markus Snellman[1], Namsoon Eom[1], Martin Ek[2], Maria E Messing[1], Knut Deppert[1]

[1]Lund University, Department of Physics and NanoLund, Box 118, 22100 Lund, Sweden

[2]Lund University, Department of Chemistry and NanoLund, Centre for Analysis and Synthesis, Box 124, 22100 Lund, Sweden

1. The spark discharge generator (SDG) setup

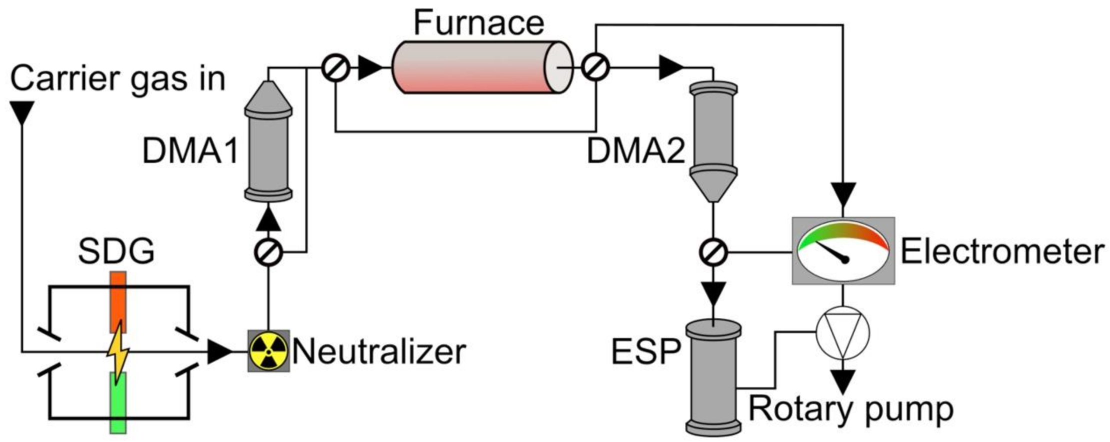

Figure S1. Schematic of the spark discharge generator (SDG) setup.

2. Size distribution measurements

Excellent control of the nanoparticles' size distribution can be obtained by deposition after size selection using a tandem DMA setup (Fig. S1). In Fig. S2, size distribution measurement on compacted CuAg filtered with an electrical mobility diameter of 30 nm is shown. Micrographs of ca. 1300 particles were acquired with a Hitachi SU8010 SEM; particle counting and equivalent circular diameter measurements were made with ImageJ. The equivalent circular diameter matches well with the electrical mobility diameter of the DMAs, and the distribution is narrow with a standard distribution of 1.35 nm as determined by a normal distribution fit. **Figure S2.** Size distribution measurement (blue histogram) and normal distribution fit (red curve) of 30 nm CuAg nanoparticles.

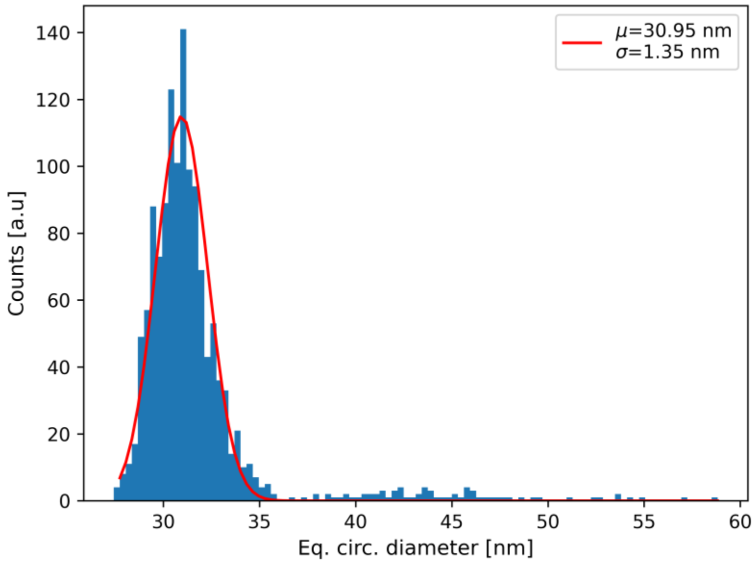

Figure S2 – Size distribution of the compacted CuAg NPs filtered with an electrical mobility diameter of 30 nm.

3. Evaporation of Ag and Cu

In the compaction study (Fig. 1), we noted a second mobility diameter plateau that we attributed to Ag evaporation, as supported by detailed STEM-EDX mappings on 3 – 4 single nanoparticles and TEM-EDX on 30 individual nanoparticles at each of the three investigated temperatures (750 °C, 850 °C, 950 °C). Here, we have estimated the evaporation of Cu and Ag using the classical Knudsen equation for evaporation rate per unit area:

$$\frac{J_{ev}}{A} = \frac{p_v}{\sqrt{2\pi MkT}} \qquad \text{(SE1)}$$

Where p_v is the vapor pressure of the pure element at temperature T, M is the molecular weight of element and k is the Boltzmann constant. Using vapor pressure data of Cu and Ag from Alcock et al. "Canadian Metallurgical Quarterly", 23 (3), 309 – 313 (1984), we have plotted J_{ev}/A for Cu and Ag in Fig. S3:

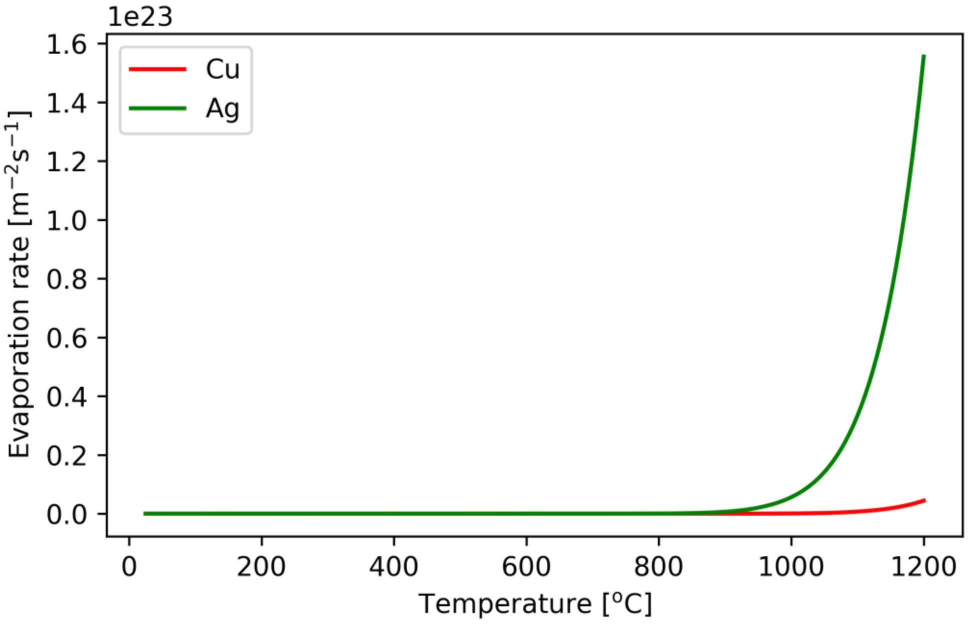

Figure S3 – Evaporation rate per unit area for pure Cu (red line) and Ag (green line).

This qualitative reasoning is in good agreement with the finding that Cu enriches in the particles due to Ag evaporation at temperatures > 900 °C.

4. NMF of STEM-EDX maps

We used non-negative matrix factorization (NMF) to segment the STEM-EDX maps to separate regions of overlapping phases and quantify the composition of these. The NMF reduces the dimensionality of the dataset to n=3 linear combinations of the original 2048 EDX channels. These new dimensions, shown in the right columns of figures S4 – S6, can be directly related to the composition of sample phases and have been used to quantify the composition of a Cu rich component and an Ag rich component. These phases' spatial extent can be visualized as loading plots, left columns in figures S4 – S6.

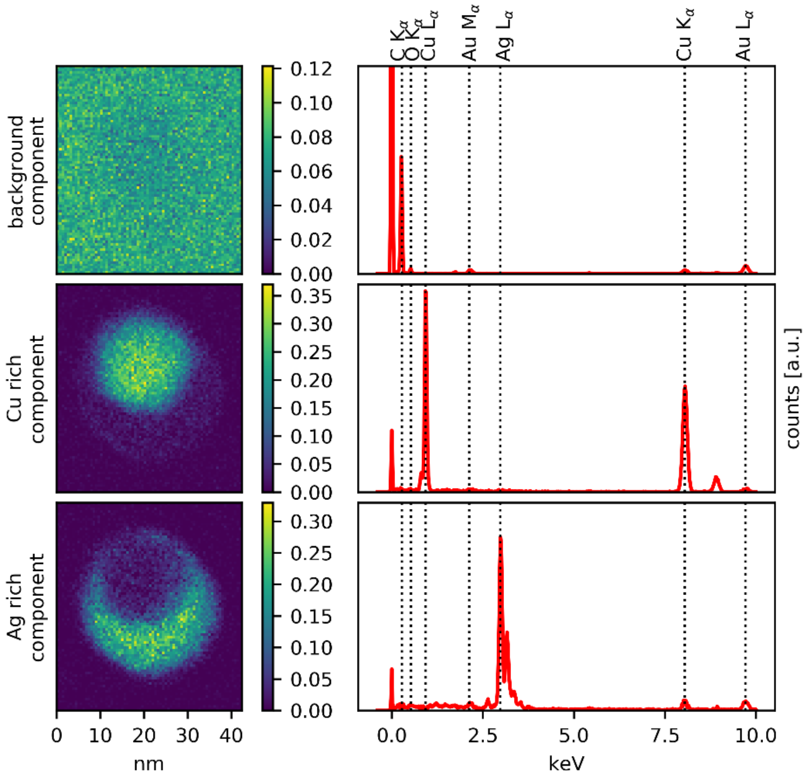

Figure S4 – NMF of STEM-EDX map of Cu@Ag particle synthesized at 750 °C. Left column, from top to bottom: loading plots of a background component, Cu rich component and Ag rich component. Right column, from top to bottom: the respective principal components from the NMF.

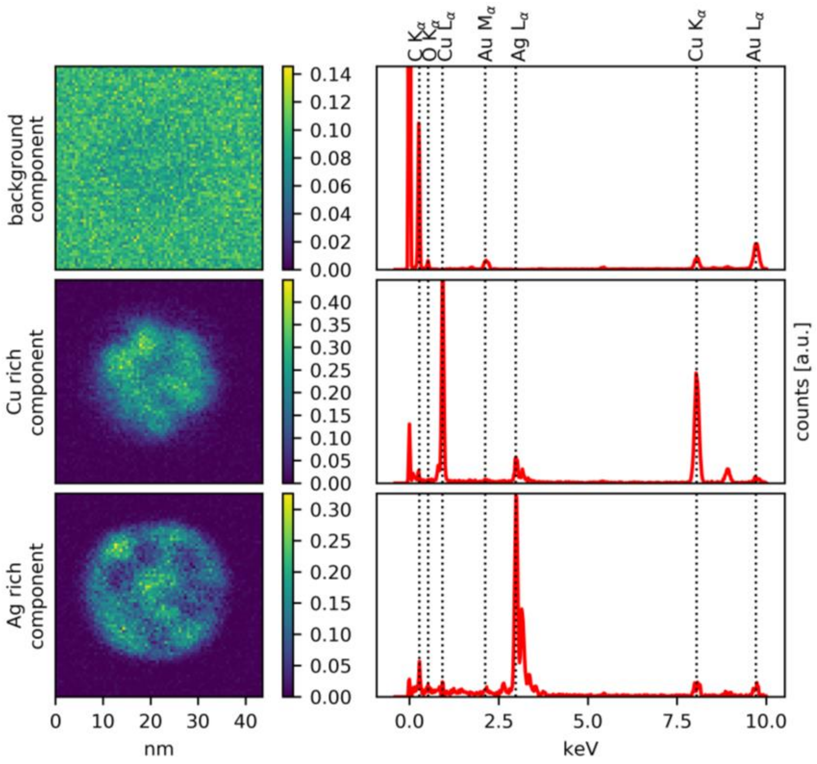

Figure S5 – NMF of STEM-EDX map of Cu@Ag particle synthesized at 850 °C. Left column, from top to bottom: loading plots of a background component, Cu rich component and Ag rich component. Right column, from top to bottom: the respective principal components from the NMF.

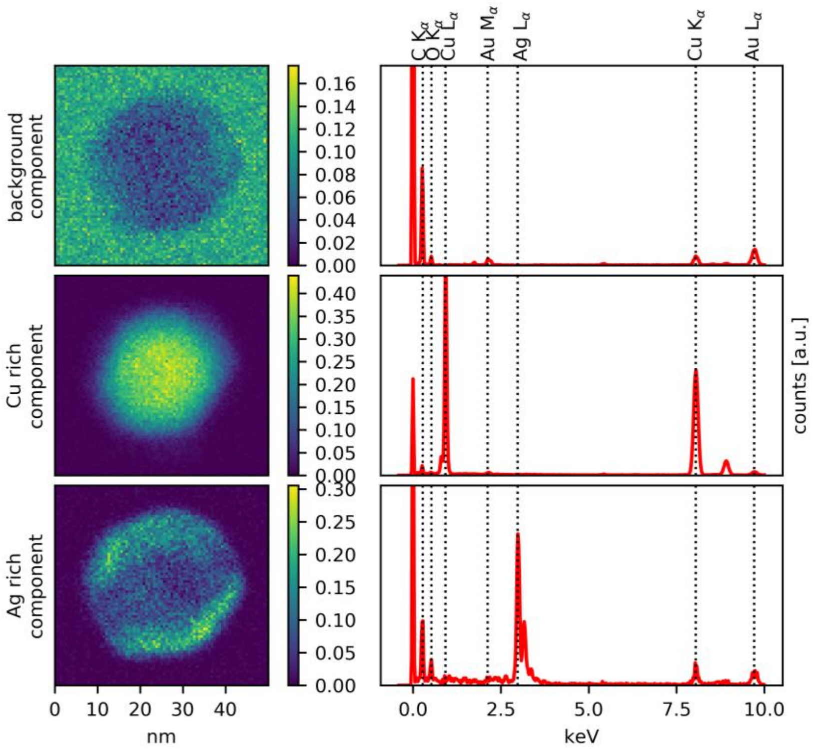

Figure S6 – NMF of STEM-EDX map of Cu@Ag particle synthesized at 950 °C. Left column, from top to bottom: loading plots of a background component, Cu rich component and Ag rich component. Right column, from top to bottom: the respective principal components from the NMF.

5. Ag shell thickness for spherical Cu@Ag core@shell nanoparticles

Consider a spherical core@shell nanoparticle with a Cu core radius of r_{core}, a Ag shell thickness of t_{shell} and a particle radius of $r_{particle}$. The volumes of the core and shell are given by $V_{core} = \frac{4\pi}{3} r_{core}^3$ and $V_{shell} = \frac{4\pi}{3}(r_{particle}^3 - r_{core}^3)$ respectively. Hence, we have

$$n_{Cu} = \frac{V_{core}}{V_{M_{Cu}}} = \frac{\frac{4\pi}{3} r_{core}^3}{V_{M_{Cu}}} \quad \text{(SE2)}$$

moles of Cu, where $V_{M_{Cu}}$ is the molar volume of Cu, and

$$n_{Ag} = \frac{V_{shell}}{V_{M_{Ag}}} = \frac{\frac{4\pi}{3}(r_{particle}^3 - r_{core}^3)}{V_{M_{Ag}}} \quad \text{(SE3)}$$

moles of Ag, where $V_{M_{Ag}}$ is the molar volume of Ag. The Ag atomic concentration can be written with equations SE1 and SE2 as

$$c_{Ag} = \frac{n_{Ag}}{n_{Cu}+n_{Ag}} = \frac{\frac{(r_{particle}^3 - r_{core}^3)}{V_{M_{Ag}}}}{\frac{r_{core}^3}{V_{M_{Cu}}} + \frac{(r_{particle}^3 - r_{core}^3)}{V_{M_{Ag}}}} \quad \text{(SE4)}$$

Which can be solved for $r_{particle}$ as

$$r_{particle} = r_{core} \sqrt[3]{\frac{\left(\frac{c_{Ag}}{V_{M_{Cu}}} - \frac{c_{Ag}}{V_{M_{Ag}}} + \frac{1}{V_{M_{Ag}}}\right)}{\frac{1}{V_{M_{Ag}}} - \frac{c_{Ag}}{V_{M_{Ag}}}}} \quad \text{(SE5)}$$

Noting that $r_{core} = r_{particle} - t_{shell}$, we can rearrange equation (SE4) to

$$t_{shell} = r_{particle} \left(1 - \frac{1}{\sqrt[3]{\frac{\left(\frac{c_{Ag}}{V_{M_{Cu}}} - \frac{c_{Ag}}{V_{M_{Ag}}} + \frac{1}{V_{M_{Ag}}}\right)}{\frac{1}{V_{M_{Ag}}} - \frac{c_{Ag}}{V_{M_{Ag}}}}}} \right) \text{(SE6)}$$

6. Supporting MD simulations

MD simulation at a lower cooling rate

To investigate the cooling rate's effect on the final mixing state of Cu-Ag nanoparticle, we conducted MD simulations at a much lower cooling rate of 0.0008 K/ps (corresponding to 1 μs for cooling). Note that the total number of atoms here is set to fewer due to the computation time.

Even at this slow cooling rate, the bimetallic system contains some of the Ag atoms in the core, and they are not uniformly mixed with Cu atoms (Figure S7).

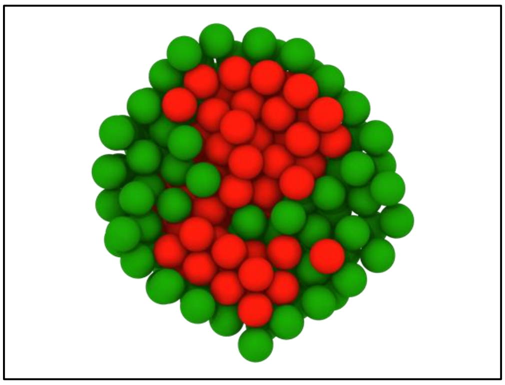

Figure S7. A Cu-Ag nanoparticle formed from a Cu(Red) with a diameter of 1.6 nm and an Ag(Green) with a diameter of 2 nm by heating up to 850 °C and cooling back to 300 K at a cooling rate of 0.0008 K/ps.

A well segregated core-shell structure

We carried out an additional MD simulation for equally sized Cu and Ag nanoparticles at a temperature of 790 °C, shown in Figure S8. At this temperature, Ag melts, but Cu does not. Thus, Ag atoms diffuse to the solid Cu nanoparticle's surface, resulting in a core-shell structure without Ag atoms in the core region.

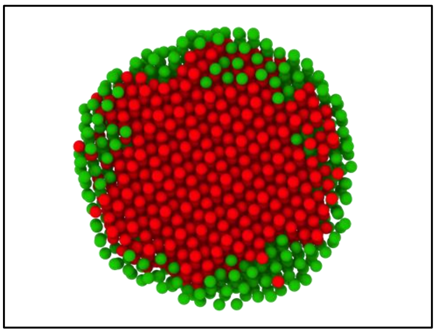

Figure S8. A Cu-Ag nanoparticle formed from equally sized Cu and Ag nanoparticles heating up to 790 °C and cooling back to 300 K. At this temperature, only Ag melts and Ag atoms diffuse to the surface of solid Cu resulting in overall core@shell morphology.

Overestimation of melting point in MD simulations

When simulations on larger particles are performed (6 nm and 10 nm in diameter), the melting temperatures are higher than that of the 4 nm ones, and thus the Janus structures form at higher temperatures. Fig S9 shows Janus-like particles formed at different sintering temperatures: Smaller particles form Janus-like structures at lower temperatures.

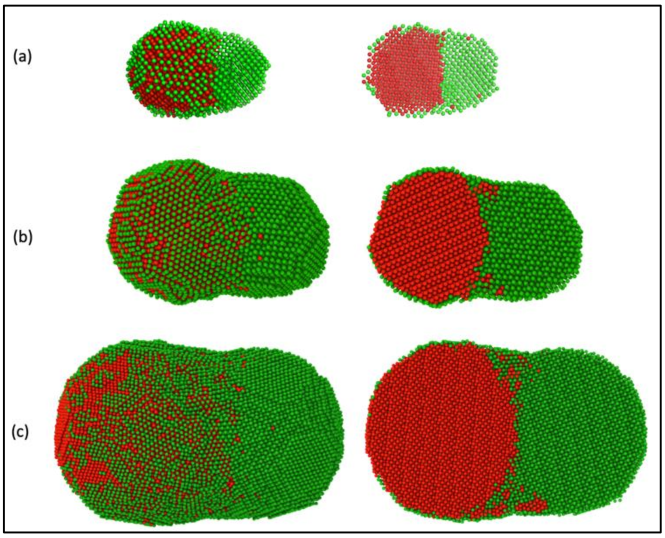

Figure S9. Janus-like structures observed in MD simulations for different Cu-Ag particle sizes. (a) For a bimetallic particle formed from Cu (3.7 nm) and Ag (3.6 nm) crystals, a Janus-like particle is formed when the system is heated to 750 °C. (b) For a bimetallic particle formed from Cu (6nm) and Ag (6 nm) crystals, a Janus-like particle is formed when the system is heated to 830 °C. (c) For a bimetallic particle formed from Cu (10 nm) and Ag (10 nm) crystals, a Janus-like particle is formed when the system is heated to 930 °C.

MD simulations for larger particles

We performed additional MD simulations for larger particles, and the results are shown in Fig S10. Here, 10 nm in diameter Cu and Ag are heated up to 930 °C and 1085 °C, and cooled to room temperature. When the system is heated up to only 930 °C, a Janus-like particle is formed and when it is heated to 1085 °C, a core@shell-like structure is formed. The trend is the same as the one for the smaller particles except for the temperatures' exact value. It appears that regardless of the particle size, Janus-like particles form at lower temperatures, and core@shell particles form at higher temperatures.

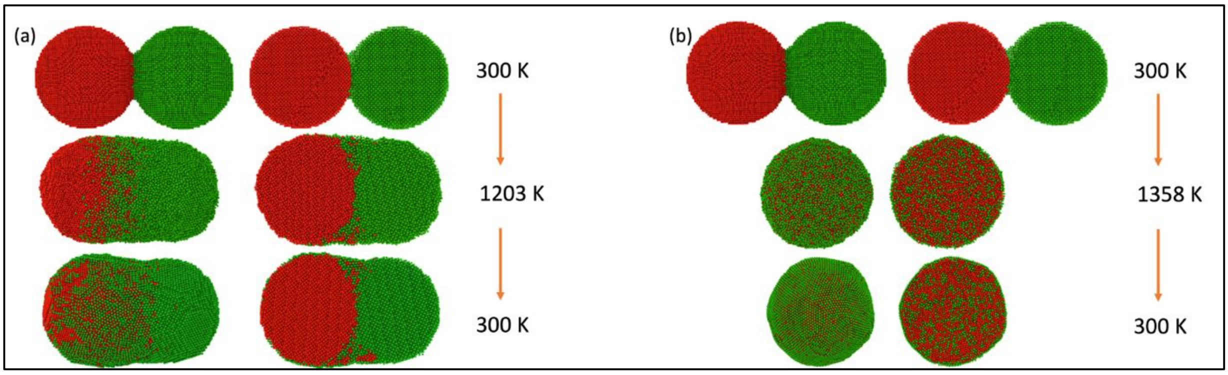

Figure S10. MD simulation results for 10 nm in diameter Cu and Ag. (a) A Janus-like structure is formed at 930 °C. (b) A Core@shell-like structure is formed at 1085 °C.
